# Immune Cell Proteins and Parkinson's Disease: A Mendelian Randomization Analysis of Causal Associations

**DOI:** 10.1002/brb3.70596

**Published:** 2025-07-09

**Authors:** Haining Li, Jianhang He, Tingting Xuan, Shue Gu, Xiaoyan Niu, Yazhou Ren, Xiuping Zhan, Jiang Cheng

**Affiliations:** ^1^ Department of Neurology General Hospital of Ningxia Medical University Yinchuan Ningxia P.R. China; ^2^ The First School of Clinical Medicine Ningxia Medical University Yinchuan Ningxia P.R. China; ^3^ Department of Neurology Wuzhong People's Hospital Wuzhong Ningxia P.R. China

**Keywords:** GWAS, immune cell proteins, MR, PD

## Abstract

**Background:**

The neuroimmune interaction mechanisms of neurodegenerative diseases have received increasing attention. Parkinson's disease (PD) is the second most common neurodegenerative disease, with potential immunoregulatory abnormalities. However, the causal roles of specific immune cell proteins remain unclear.

**Methods:**

We obtained PD and immune cell protein data from an open and free genome‐wide association study (GWAS) for subsequent analysis. Two‐sample MR analyses with inverse‐variance weighted (IVW), MR‐Egger regression, weighted median, and weighted mode methods were used to evaluate the causal effects. Sensitivity analyses incorporated Cochran's Q test for SNP heterogeneity (prioritizing IVW estimates when present) alongside MR‐Egger intercept and leave‐one‐out evaluations to address horizontal pleiotropy.

**Results:**

The IVW analysis revealed that the genetically predicted level of three immune cell proteins per standard‐deviation increase was positively associated with PD, including CD38 (OR = 1.13, 95%CI: 1.05–1.22, *P* = 0.001), FcγRIIIB (OR = 1.06, 95%CI: 1.01–1.11, *P* = 0.019), and CUL4B (OR = 1.11, 95% CI: 1.00–1.20, *P* = 0.012). The IVW analysis also revealed that the genetically predicted level of ADAMTSs per standard‐deviation increase was inversely associated with PD (OR = 0.89, 95% CI: 0.81–0.98, *P* = 0.013).

**Conclusions:**

We demonstrate that CD38, FcγRIIIB, and CUL4B are risk factors for PD, whereas ADAMTSs is a protective factor.

## Graphical abstract

Mendelian randomization (MR) analysis was applied to elucidate the causal relationship between immune cell proteins and PD. Our study reveals a possible causal effect of immune cell proteins on the risk of PD and provides new ideas for the prevention and management of PD through immune cell proteins.

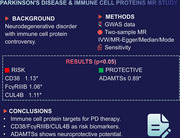

## 1 Introduction

PD is the second most common neurodegenerative disease globally, imposing an increasingly heavy burden on public health. It is characterized by progressive dopaminergic neuron degeneration in the nigrostriatal pathway and clinically presents with debilitating motor dysfunction (Jankovic 2008). Due to the overlapping PD phenotypes and the lack of objective biomarkers, the diagnosis remains uncertain, leading to delayed interventions and soaring medical costs. Increasing evidence suggests that neuroimmune interactions are involved in the pathogenesis of PD. However, the causal relationship between specific immunomodulatory factors and neuronal degeneration remains mechanistically unclear. This study utilizes the framework of genetic epidemiology to systematically evaluate the possibility of immune‐related protein biomarkers as potential drivers of PD progression. The aim is to identify effective targets for early therapeutic intervention and promote biomarker‐based diagnostic innovation.

The pathogenesis of PD is complex, involving multiple factors such as genetics, physical constitution, living environment, and lifestyle (Zhichun Chen et al. 2020; Raza et al. 2019). Multiple factors, including mitochondrial dysfunction, oxidative stress, protein aggregation, autophagy impairment, and neuroinflammation, significantly influence different patients (Simon et al. 2020; Subramaniam and Chesselet 2013). Recent research has emphasized the potential for maladaptive immune and inflammatory responses originating in the gastrointestinal tract. These mechanisms may accelerate the pathogenesis of PD (Morris, Spillantini, Sue, and Williams‐Gray 2024). Among these factors, chronic neuroinflammation can contribute directly or indirectly to the onset and progression of PD (Zhihong Chen and Trapp 2016). The inflammatory mediators associated with neuroinflammation include proteins, chemokines, cytokines, and cytokines. Immune cells and their related proteins may play a crucial role in the pathophysiological process of PD. Numerous clinical studies and animal models have demonstrated the involvement of inflammatory mediators in PD, including interferon‐gamma receptor 1 (IFNGR1) (Yan et al. 2023), Podocalyxin (S.‐J. Chen et al. 2019; Sudhaman et al. 2016), DKK1 (Lin et al. 2021; Vlasov et al. 2021), CD38 (Garfias et al. 2019; Ge et al. 2023; Maple‐Grødem et al. 2023; S. Zhou et al. 2023), HLAII histocompatibility antigen DQ α 2 chain (HLA‐DQA2) (Hill‐Burns et al. 2011), sepiapterin reductase (SPR) (Tobin et al. 2007), and estrogen receptor (ER) (Palacios et al. 2010). These molecules regulate the activity of microglia through NF‐kB, Jak/Stat, TLR, and other pathways, and actively participate in the pathophysiological processes related to PD. Results from a multicenter cohort study showed that elevated NLRP3 levels were associated with the status of PD (Panicker et al. 2022). Moreover, inhibitors targeting immune cell proteins have provided novel insights and strategies for the prevention and treatment of PD. To date, the reported research results have shown inconsistencies and limitations. The causal relationship between immune cell proteins and PD remains unknown. Our study employs two‐sample MR to establish causal links between immune cell proteins and PD risk, and identify biomarkers for early diagnosis.

Randomized clinical trials (RCTs) are widely recognized as the gold standard for proving causality. However, ethical and other factors limit their application, while other observational clinical studies are frequently affected by unmeasured confounding and reverse causation, complicating causal inference between exposure and outcome. In the absence of high‐quality RCTs, alternative study designs are urgently needed to assess causal relationships. MR offers a promising solution to these challenges. In contrast to traditional observational studies, MR analysis overcomes various limitations, including issues related to reverse causation, measurement errors, and underlying biases. A significant advantage of the MR approach is the use of genetic variants as proxy variables for each trait. In recent studies, researchers have employed MR to examine the association between the SPR gene, the CAM1E469K polymorphism (Sharma et al. 2011), and the risk of PD. However, very few studies have fully explored the causal relationship between immune cell proteins and PD pathogenesis using MR methods.

Based on GWAS data, this study aims to explore the causal relationship between immune cell proteins and PD at the genetic level through MR. The results of this study will help to reveal the roles of immune cell proteins in the pathogenesis of PD, and provide new ideas for future diagnosis and treatment.

## 2 Materials and Methods

### 2.1 Study Design

**FIGURE 1:**
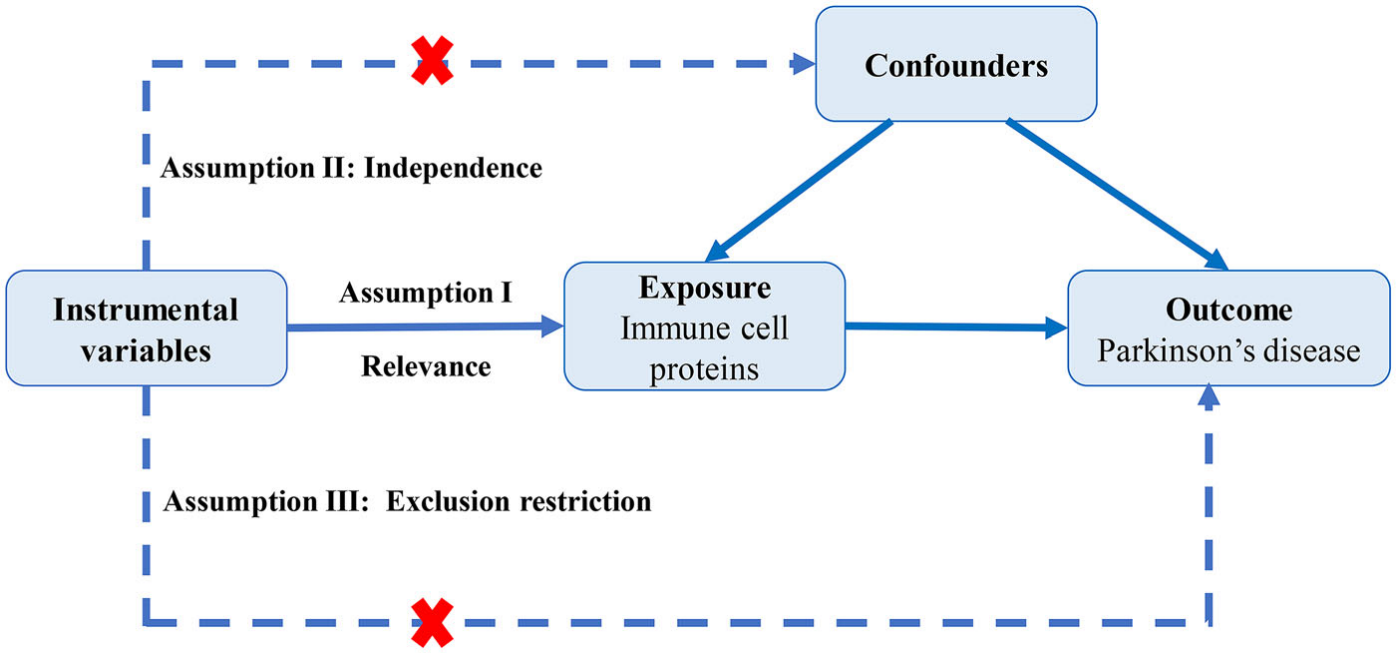
Three basic assumptions in MR analysis.

MR uses genetic variations as instrumental variables (IVs) to infer the causal relationship between exposure factors and outcomes. By leveraging the random allocation of alleles during gametogenesis, it minimizes the confounding effects caused by post–birth environmental or behavioral factors. In order to ensure robust causal inference in our investigation of immune cell proteins' role in PD, we implemented stringent IV selection criteria grounded in MR's core assumptions: (1) The selected IVs must be highly correlated with exposure, with *P* < 1 × 10‐5 as the strong correlation standard. In our analysis, the *F* statistic was used to evaluate the strength of the IV–exposure correlation. *F* is expressed as *R^2^* (*n*—*k* ‐ 1)/ [*k* (1 ‐ R^2^)]. In this equation, *R^2^* refers to the cumulative explained variance of selected SNPs on circulating immune cell protein levels, *k* is the number of selected SNPs and n is the sample size. If *F* > 10, the correlation was perceived as strong enough to avoid the bias caused by weak IVs; (2) the selected IVs must not be associated with any confounding factors of the exposure–outcome relationship; and (3) the IVs must only affect the outcome through the exposure (Figure 1). These criteria were specifically optimized for studying complex protein–disease relationships. By selecting instrumental variables based on the principles of genetic epidemiology, our method improves the reliability of identifying immune mediators with true causal relationships in the pathogenesis of PD, and provides a template for biomarker validation studies of neurodegenerative diseases.

### 2.2 Data Sources

The pertinent data emanate from 24 distinct GWAS databases (Table S1). The original studies secured informed consent from the participants. Our study was exempted by the ethics committee because the original study signed an informed consent form, and this study was a second analysis.

### 2.3 Screening for SNPs

Significant SNPs were screened from the GWAS summary data of each type of protein (with *P* < 1 × 10^−5^ as the screening condition, assumption 1); the chain disequilibrium coefficient r^2^ was set to be 0.001, and the width of chain disequilibrium region was set to be 10,000 kb to ensure that the individual SNPs were independent of each other and to exclude the influence of gene pleiotropy on the results; and SNPs were eliminated by PhenoScanner that were associated with confounders and SNPs associated with outcome (hypotheses 2 and 3). The relevant SNPs screened above were extracted from the summarized GWAS data of Parkinson's disease; a minimum *R^2^* > 0.8 was set. information from the above dataset was summarized, and SNPs directly related to PD were also excluded (*P* < 1 × 10^−5^). Out of the 495 selected SNPs, we collected information on the effect allele (EA), EA frequency, effect sizes, standard error (SE), and *P*‐value. For the outcome datasets, GWAS data on PD were acquired from an independent GWAS analysis, encompassing 482,730 cases with European ancestry (Table S2).

### 2.4 Causality Analysis

For causality analysis, we used IVW, MR‐Egger regression, weighted median estimation (WME), and weighted model methods to evaluate the causal effects of immune cell proteins on PD. The IVW is the most important causal analysis method, which does not require individual‐level data and allows the causal effect value to be calculated directly using the collected data. While MR‐Egger regression and weighted median estimator are used as auxiliary causal analysis methods.

### 2.5 Heterogeneity and Sensitivity Test

The sensitivity analysis included heterogeneity and pleiotropy. The heterogeneity of SNPs was assessed using Cochran's Q test, with a focus on the results of the IVW model if heterogeneity was present. Pleiotropy analysis utilized the intercept term of the MR‐Egger method. We conducted a leave‐one‐out sensitivity analysis to assess the stability of the effect sizes and to identify any individual SNP that might disproportionately influence the association. This involved sequentially removing each SNP and applying the IVW method to the remaining SNPs.

### 2.6 Statistical Analysis

We harmonized the exposure and outcome datasets to ensure that the exposure alleles (EAs) consistently corresponded to the same allele. All these methodologies were implemented using the TwoSampleMR package in the R 4.2.2 software, with a significance level set at α = 0.05. The Benjamini–Hochberg procedure implemented in R 4.2.2 was used to obtain adjusted *P*‐values; *P* < 0.05 was considered statistically significant in genetic correlation and MR analyses.

## 3 Results

### 3.1 Genetic Variant Selection

Following multiple screenings, 495 SNPs were ultimately included in the PD dataset (Table S2). F statistic greater than 10 was used as an index to evaluate weak instrumental variables. The distribution of an *F*‐statistics for individual SNPs ranged from 19.507 to 1570.604, with a mean value of 26.045. The range and mean of the distribution of the *F*‐statistic suggest that causal associations are unlikely to be confounded by weak instrumental variables.

### 3.2 TSMR Analysis

**FIGURE 2:**
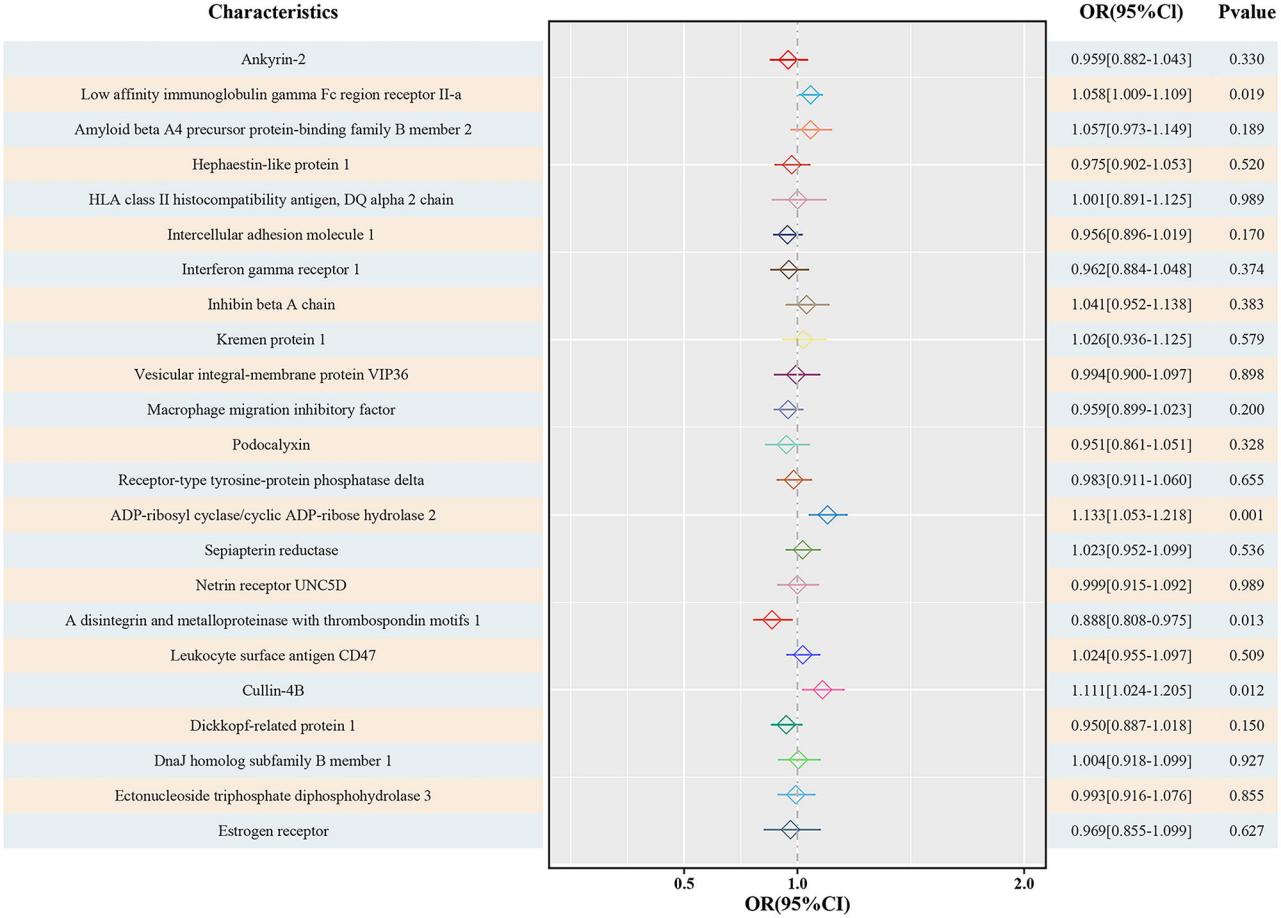
MR results.

The results of the TSMR analysis are presented in Table 1. Our findings suggested that CD38, FcγRIIIB, and CUL4B are risk factors for PD, whereas ADAMTSs are protective factors for PD. The IVW analysis revealed that the genetically predicted level of three immune cell proteins per standard‐deviation increase was positively associated with PD, including CD38 (OR = 1.13, 95%CI: 1.05–1.22, *P* = 0.001), FcγRIIIB (OR = 1.06, 95%CI: 1.01–1.11, *P* = 0.019), and CUL4B (OR = 1.11, 95% CI: 1.00–1.20, *P* = 0.012) (Figure 2). In addition, except for CUL4B, the results of MR‐Egger, WME, and weighted‐mode methods of other immune cell proteins showed consistent results (Table 1). The IVW analysis also revealed that the genetically predicted level of ADAMTSs per standard‐deviation increase was inversely associated with PD (OR = 0.89, 95% CI: 0.81–0.98, *P* = 0.013) (Figure 2). The causal effects obtained by MR‐Egger, WME, and weighted mode methods were not completely consistent, but combined with the multiple results (*P* > 0.05), the IVW results were finally obtained. Our research is an exploratory study. After FDR correction, we found that CD38 still showing a causal effect on PD. In addition, there were three immune cell proteins (FcγRIIIB, CUL4B, and ADAMTSs) showed a causal effect on PD, although no significant association between immune cell proteins and PD was detected after FDR correction (Table 1). Besides, the results of the IVW estimates showed that genetically predicted levels of ANK2 and 19 other immune cell proteins were not associated with the risk of PD (*P* < 0.05) (Table S3).

**TABLE 1:** Four MR regression causal correlation results.

Exposures	SNPs	Methods	OR	95%CI	*P‐*value	*P*_FDR
CD38	23	MR Egger	1.16	1.03–1.29	0.021	0.246
		Weighted median	1.24	1.13–1.35	0.000	0.000
		Inverse variance weighted	1.13	1.05–1.22	0.001	0.018
		Weighted mode	1.23	1.12–1.34	0.000	0.005
FcγRIIIB	31	MR Egger	1.09	1.02–1.17	0.013	0.294
		Weighted median	1.08	1.03–1.13	0.001	0.006
		Inverse variance weighted	1.06	1.00–1.11	0.019	0.109
		Weighted mode	1.08	1.03–1.13	0.002	0.018
CUL4B	22	MR Egger	1.15	0.94–1.39	0.185	0.531
		Weighted median	1.07	0.96–1.20	0.215	0.988
		Inverse variance weighted	1.11	1.02–1.20	0.012	0.133
		Weighted mode	1.08	0.91–1.28	0.396	0.910
ADAMTSs	16	MR Egger	0.96	0.80–1.16	0.700	0.847
		Weighted median	0.89	0.79–1.01	0.073	0.562
		Inverse variance weighted	0.89	0.81–0.98	0.013	0.098
		Weighted mode	0.90	0.74–1.09	0.300	1.000

*Note*: The first column gives the names of the immune cell proteins (CD38: ADP‐ribosyl cyclase/cyclic ADP‐ribose hydrolase 2; FcγRIIIB: Low affinity immunoglobulin gamma Fc region receptor II‐a; CUL4B: Cullin‐4B; ADAMTSs: A disintegrin and metallPDroteinase with thrombospondin motifs 15). The Sixth and Seventh columns list the unadjusted (‐value) and adjusted *P*‐values (*P*_FDR) according to Benjamini/Hochberg (BH).

### 3.3 Heterogeneity Test

**FIGURE 3:**
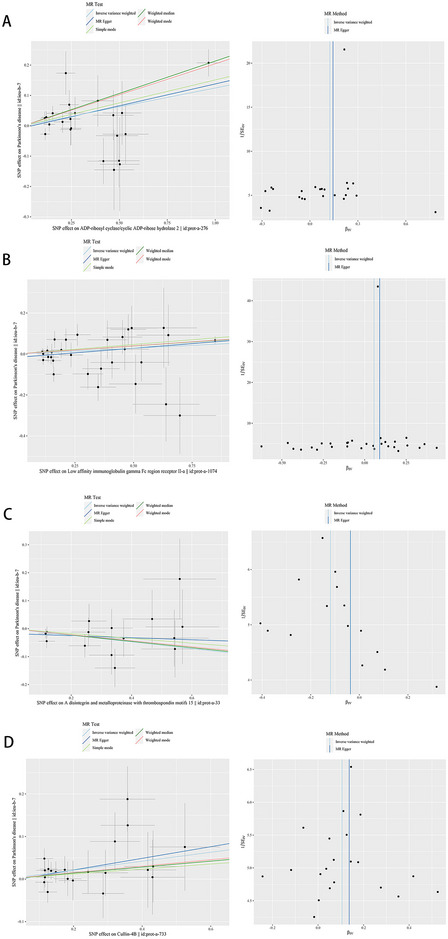
A plot relating the effect sizes of the SNP–CD38(A) FcγRIIIB; (B) CUL4B; (C) ADAMTSs; and (D) association (x‐axis and log OR) and the SNP‐PD associations (y‐axis and log OR) with the standard error bars. The slopes of the lines correspond to causal estimates using each of the four different methods (weighted median, weighted mode, IVW random effects, and MR‐Egger); Funnel plot showing the relationship between the causal effect of CD38(A) FcγRIIIB; (B) CUL4B; (C) ADAMTSs; and (D) on PD estimated using each individual SNP as a separate instrument against the inverse of the standard error of the causal estimate. Vertical lines show the causal estimates using all SNPs combined into a single instrument for each of the two different methods (IVW random effects, and MR‐Egger). There is no significant asymmetry in the plot.

Cochran's Q test of the IVW method indicated that no significant heterogeneity in the estimation of included SNPs in CD38–PD, CUL4B–PD, and ADAMTSs–PD (*P* = 0.079, *P* = 0.839, *P* = 0.792) (Table S3). The result of the FcγRIIIB‐PD test showed heterogeneity (*P* < 0.05). The MR‐Egger regression intercept term showed no statistically significant difference from zero (*P* = 0.661, *P* = 0.176, *P* = 0.729, *P* = 0.321), leading to the conclusion that there was no genetic pleiotropy among the SNPs (Table S3). The funnel plot results for CD38, FcγRIIIB, CUL4B, and ADAMTSs showed good symmetry and did not show any outliers or outliers on the scatterplot (Figure 3), implying that potential bias had minimal impact on causal associations and that the MR analysis results were highly robust, among others.

### 3.4 Sensitivity Analyses

**FIGURE 4:**
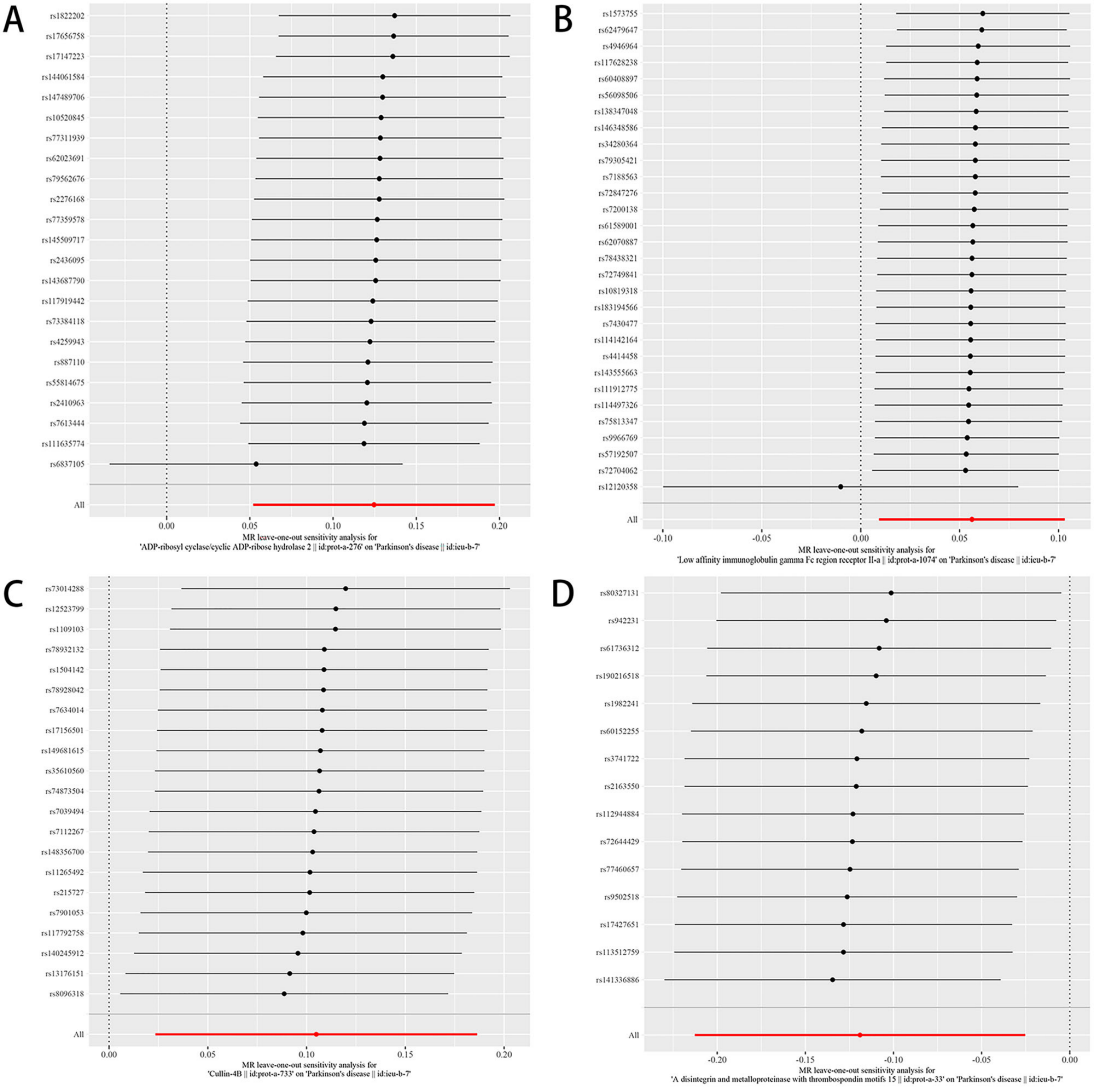
MR leave‐one‐out sensitivity analysis for CD38, FcγRIIIB, CUL4B, ADAMTSs in PD. Each black point represents the IVW MR method applied to estimate the causal effect of CD38, FcγRIIIB, CUL4B, ADAMTSs in PD, excluding that particular variant from the analysis. The red point depicts the IVW estimate using all SNPs. There are no instances where the exclusion of one particular SNP leads to dramatic changes in the overall result.

Sensitivity analyses using the leave‐one‐out method indicated that the results with the exclusion of each CD38, FcγRIIIB, CUL4B, and ADAMTSs associated SNP sequentially were similar to those with the inclusion of all SNPs, and no SNP exhibited a substantial impact on causal association estimates (Figure 4). These findings affirm the robustness of the MR results in this study.

This MR study identified CD38, FcγRIIIB, and CUL4B as PD risk factors and ADAMTSs as protective, with robust IVs validated through sensitivity and heterogeneity tests. Consistent results across methods and absence of pleiotropic bias highlight the biological relevance of these immune cell proteins in PD pathogenesis.

## 4 Discussion

The pathogenesis of PD involves multiple mechanisms, including oxidative stress, excitotoxicity, mitochondrial dysfunction, impaired autophagy, and neuroinflammation (Gan, Cookson, Petrucelli, and La Spada 2018; Ransohoff 2016). Recent evidence suggests that neuroinflammation is a core driving factor in the progression of PD, characterized by microglial activation (Isik, Yeman Kiyak, Akbayir, Seyhali, and Arpaci 2023; Kwon and Koh 2020), astrocytic reactivity in nigrostriatal pathways, systemic inflammatory markers (Gopinath, Mackie, Phan, Tansey, and Khoshbouei 2023), and genetic polymorphisms (Pan et al. 2023) in immune‐related PD risk loci (such as LRRK2, SNCA) (Li, Tan, and Yu 2014; Sliter et al. 2018). Although observational studies have suggested that immune dysregulation is involved in the pathogenesis of PD (Hirsch and Hunot 2009), establishing causal relationships between specific immune cell proteins and disease risk remains challenging due to confounding factors. This study explores the causal relationship between immune cell proteins and PD and highlights potential molecular targets, providing valuable insights for early diagnosis and identification of potential drug targets.

CD38 is a transmembrane glycoprotein consisting of a short cytoplasmic tail, a transmembrane domain, and an extracellular domain (Malavasi et al. 2008). It catalyzes nicotinamide adenine dinucleotide (NAD+), which mobilizes intracellular calcium stores and regulates astrocyte maturation as well as oligodendrocyte precursor cell differentiation (Hogan et al. 2019). Decreased NAD^+^ levels are common in PD (Guerreiro et al. 2020). NAD^+^ is a potent neuroprotective and anti‐inflammatory agent (Lautrup et al. 2019), and it is hypothesized that NAD^+^ levels are regarded as a negatively correlated marker of CD38 expression and activity (Camacho‐Pereira et al. 2016). This indirectly suggests increased CD38 expression in PD, potentially contributing to neurodegeneration and neuroinflammation. A recent Mendelian randomization (SMR) analysis based on pooled data showed that CD38 exhibited a significant association with PD at both protein and mRNA levels (Shi et al. 2024). This suggests that CD38 may predispose individuals to PD, consistent with the findings of this study. Some experimental data suggest that CD38 knockout mice are protected from neurodegenerative diseases and neuroinflammation (Kou et al. 2009; Ma et al. 2014). Inhibition of CD38 and supplementation with nicotinamide riboside (NR) increases NAD^+^ levels, which in turn inhibits the NF‐κB signaling pathway in microglia and suppresses neuroinflammation in the brain (Roboon et al. 2021). Given that CD38 regulates the bioavailability of NAD^+^ in the brain and the activity of NAD‐dependent enzymes essential for neuronal survival, enhancing NAD^+^ levels could be crucial in treating PD. In addition to its many biological activities, apigenin has been reported to be a neuroprotective agent (Patil et al. 2022). Apigenin has shown anti‐neuroinflammatory effects in preclinical studies, indicating that it has neuroprotective effects against neuronal degeneration in certain neurodegenerative diseases (Olasehinde and Olaokun 2024). In a rotenone‐induced Parkinson rat model, tyrosine hydroxylase (TH) immunoreactivity is lost in the striatum and substantia nigra, and apigenin treatment reduces alpha‐synuclein aggregation and increases TH protein expression and dopamine D2 receptor (D2R) expression (Charrière et al. 2024). In addition, apigenin crosses the blood‐brain barrier (BBB) and increases NAD^+^ concentration in the brain to exert a neuroprotective effect. Weight changes, dyskinesia, oxidative stress, neuroinflammation, and neurotransmission were significantly reduced in the brains of lipopolysaccharide (LPS)‐induced PD rats after treatment with apigenin alone or in combination with piperine (Patel and Singh 2022). Despite the promising applications of apigenin in PD, further studies are needed to elucidate the exact molecular mechanisms of apigenin's action and to assess its safety and efficacy in human populations.

FcγRIIIB may be a potential risk factor for PD, and no studies have been found to correlate FcγRIIIB with PD. FcγRIIIB is a receptor highly expressed on neutrophils that binds to the Fc portion of immunoglobulins and plays a key role in regulating neutrophil recruitment and homologous clearance of immune complexes (Gillis et al. 2014). Previous studies have suggested that the peripheral immune system may be involved in the neuroinflammation associated with PD (Song et al. 2024). It is involved in the pathogenesis of PD mainly through the mechanisms of inflammatory response, cellular infiltration, dysregulation of lymphocyte subsets, and autoimmune reactions. Neutropenia, lymphopenia, and monocytopenia were observed in PD (Hakimi et al. 2011). We therefore hypothesized that increased FcγRIIIB expression may be involved in the pathogenesis of PD by mediating inflammatory responses, recruiting neutrophils, and releasing pro‐inflammatory mediators, but the pathogenic mechanism remains unclear. Increased FcγRIIIB expression may increase the risk of developing PD, a possibility that remains to be verified by larger MR studies as well as by clinical trials.

CUL4B may be a risk factor for PD. Fewer studies have been conducted on the causal relationship between CUL4B and PD, and only one multilayer bioinformatics analysis was found showing that CUL4B expression was significantly increased in PD (Yao et al. 2022), which is consistent with our study and suggests that CUL4B may increase the risk of PD. CUL4B is a scaffolding protein in the Cullin‐4B‐Ring E3 ligase complex (CRL4B). It is involved in the regulation of various physiological and developmental control processes by targeting specific substrates for ubiquitin‐dependent degradation or modification. Apoptosis is an important mechanism in embryonic development and maintenance of tissue homeostasis (Kerr, Wyllie, and Currie 1972). If apoptosis is dysregulated, a variety of diseases, such as neurodegenerative diseases and cancer may occur. Previous studies have shown that CLU4B may be involved in apoptosis by targeting mediated ubiquitination of apoptotic protease activator 1 (Apaf‐1), and ubiquitinated Apaf‐1 activates caspase‐9 under conditions of proteasomal damage. Caspase‐9, as one of the cysteine protease family, is also a pathway and enhances programmed cell death when activated (Ohta et al. 2019). In neurodegenerative diseases, such as multiple system atrophy (Kawamoto et al. 2016), Huntington's disease (Kiechle et al. 2002), and Alzheimer's disease (Rohn et al. 2002), several studies have shown that total and cleaved expression of caspase‐9 is increased in apoptotic neurons, glial cells, serum, and cerebrospinal fluid. Although not yet reported in PD, we hypothesize that CUL4B‐mediated activation of caspase‐9 by Apaf‐1 ubiquitination results in activation of the nigrostriatal apoptotic cascade, which leads to degenerative death of the nigrostriatum and ultimately PD. At present, the pathogenic mechanism of CUL4B in PD is still unclear and requires further research, and larger MR studies are necessary.

ADAMTSs may be a potential protective factor in preventing PD. Previous studies have shown that changes in the expression and activity of ADAMTSs are associated with neurodegenerative diseases, stroke, motor neuron disease, schizophrenia, and even Alzheimer's disease (Lemarchant et al. 2017). ADAMTSs are involved in a variety of physiological and pathological processes, including degradation and remodeling of extracellular matrix components, inhibition of angiogenesis, and regulation of inflammatory processes (Lemarchant et al. 2013). These characteristics may contribute to the repair of neurodegenerative diseases, but the pathogenesis is still unclear. In addition, ADAMTSs can play an active role in neuronal germination, synaptic remodeling, and synaptic formation (Gottschall and Howell 2015; Mayer et al. 2005), which makes ADAMTSs very important for the recovery and repair of neuronal and/or synaptic loss. At present, it is believed that the pathological changes of PD and the non‐physiological stimulation of dopamine receptors are the main causes of synaptic structure and function abnormality (synaptic plasticity). We hypothesized that ADAMTSs have a neuroprotective effect on PD by acting on striatal nerves and/or synapses to repair synaptic transmission and induce axonal elongation. Axon elongation may be related to the induction of thrombin‐reactive protein repeat sequence of ADAMTSs, because the expression of inactive‐form proteolytic enzyme can stimulate axon growth as effectively as the active‐form proteolytic enzyme (Hamel et al. 2008). Excitingly, a recent study has for the first time identified a critical role for the ADAMTS8 gene in hypothalamic structural morphology and reported a strong genetic link to a variety of neuropsychiatric traits and diseases, including PD, with MR analyses suggesting a potential causal association between lower levels of ADAMTS8 expression and greater hypothalamic volume (S.‐D. Chen et al. 2024). Previous studies have shown that patients with PD have a significantly reduced area of hypothalamic nucleus volume, which may be due to the destruction of dopamine neurons in the hypothalamus, leading to a reduction in the number of neurons (Breen et al. 2016). Furthermore, it has also been shown that despite the large amount of histopathologic evidence of hypothalamic involvement in PD (Dayan et al. 2018), it seems to be undetectable by MRI‐based volumetric quantification (Gorges et al. 2019). However, a recent study of a fully automated hypothalamic segmentation tool based on deep convolutional neural networks has shown that microstructural changes in the hypothalamus can be detected already in the early stages of neurodegeneration in PD (Dayan and Sklerov 2021; C. Zhou et al. 2024). We hypothesize that ADAMTS8 may contribute to PD by acting on the microstructure of the hypothalamus, and although ADAMTS8is a member of the ADAMTSs family, it does suggest that ADAMTSs is indeed associated with PD and warrants further investigation. Our research showed that ADAMTSs may be a potential protective factor for PD, but further research and clinical verification are needed.

Our study possesses multiple strengths. Primarily, it represents the inaugural MR investigation examining the causal links between 23 immune cell proteins and PD utilizing a comprehensive summary of the latest data. Unlike traditional observational studies, which are frequently prone to reverse causation and confounding, MR studies inherently prevent reverse causation and substantially reduce residual confounding, thereby enhancing the credibility of the findings. Moreover, we utilized several alternative methodologies to assess the causal relationships among these factors, consistently obtaining effect estimates that affirm the robustness of our results.

However, we need to recognize certain limitations of our study. Firstly, our study predominantly included participants of European descent, potentially limiting the generalizability of our conclusions to diverse populations. Secondly, the reliability of the MR study hinges on the robustness of the initial genome‐wide association study data, potentially confining the results to genetic associations rather than establishing causality. Therefore, these findings should be interpreted with caution, and additional clinical studies are essential to validate and elucidate these relationships. Thirdly, due to data limitations, our study did not examine specific indicators, offering only a preliminary outline of their causal relationship, which necessitates further research for refinement. Fourthly, it is imperative to recognize that SNP phenotypes related to immune cell proteins and PD data are influenced by environmental factors, genetic background, and age at exposure. For instance, the incidence of PD may vary based on risk factors (such as pesticide exposure) and protective factors (such as physical activity and smoking habits) (Song et al. 2024). Consequently, accounting for these potential biases is crucial when interpreting the genetic findings.

## 5 Conclusion

CD38, FcγRIIIB, and CUL4B are identified as risk factors for PD, whereas ADAMTSs serve as a protective factor. Our study comprehensively investigated the pathogenesis of PD, pinpointed potential molecular targets, and proposed directions for the development of targeted therapeutic drugs for PD. Additionally, more extensive research and larger sample sizes are urgently needed to thoroughly explore these associations and corroborate our findings through comprehensive genetic and environmental investigations.

## Author Contributions

**Haining Li**: writing–review and editing, visualization, methodology. **Jianhang He**: writing–original draft, writing–review and editing, data curation, visualization. **Tingting Xuan**: methodology, formal analysis. **Shue Gu**: conceptualization, methodology. **Xiaoyan Niu**: soft‐ware, writing–review and editing. **Yazhou Ren**: conceptualization. **Xiuping Zhan**: funding acquisition, supervision, resources. **Jiang Cheng**: software, investigatioin, project administration, writing–review and editing.

## Conflicts of Interest

The authors declare no conflicts of interest.

## Peer Review

The peer review history for this article is available at https://publons.com/publon/10.1002/brb3.70596

## Supporting information



Table S1 Brief information of GWAS database in MR research

Table S2

Table S3 Associations of multiple proteins with PD in Univariable MR analysis

## Data Availability

The GWAS summary statistics used to perform the analyses described in the study were obtained from publicly available published data. All data generated or analyzed in the study were included in the article and supporting information.
