# Supplementary material for: Immune Cell Proteins and Parkinson's Disease: A Mendelian Randomization Analysis of Causal Associations
Source: Brain Behav. 2025 Jul 9;15(7):e70596. doi: 10.1002/brb3.70596 (PMC12241703; doi:10.1002/brb3.70596)
Supplement: Supplementary file 1 — Table S1 Brief information of GWAS database in MR research [file BRB3-15-e70596-s001.docx]

| **Table S1** Brief information of GWAS database in MR research | | | | | | |
| --- | --- | --- | --- | --- | --- | --- |
| **Exposure** | **Nsample** | **Nsnp** | **PPDulation** | **Database** | **Sex** | **Year** |
| ANK2 | 3301 | 10,534,735 | European | IEU PDen GWAS project | Male and female | 2018 |
| FcγRⅢB | 3301 | 10,534,735 | European | IEU PDen GWAS project | Male and female | 2018 |
| FE65 | 3301 | 10,534,735 | European | IEU PDen GWAS project | Male and female | 2018 |
| HEPHL1 | 3301 | 10,534,735 | European | IEU PDen GWAS project | Male and female | 2018 |
| HLA-DQA2 | 3301 | 10,534,735 | European | IEU PDen GWAS project | Male and female | 2018 |
| ICAM-1 | 3301 | 10,534,735 | European | IEU PDen GWAS project | Male and female | 2018 |
| IFNGR1 | 3301 | 10,534,735 | European | IEU PDen GWAS project | Male and female | 2018 |
| INHBA | 3301 | 10,534,735 | European | IEU PDen GWAS project | Male and female | 2018 |
| Kremen1 | 3301 | 10,534,735 | European | IEU PDen GWAS project | Male and female | 2018 |
| VIP36 | 3301 | 10,534,735 | European | IEU PDen GWAS project | Male and female | 2018 |
| MIF | 3301 | 10,534,735 | European | IEU PDen GWAS project | Male and female | 2018 |
| Podocalyxin | 3301 | 10,534,735 | European | IEU PDen GWAS project | Male and female | 2018 |
| PTRRD | 3301 | 10,534,735 | European | IEU PDen GWAS project | Male and female | 2018 |
| CD38 | 3301 | 10,534,735 | European | IEU PDen GWAS project | Male and female | 2018 |
| SPR | 3301 | 10,534,735 | European | IEU PDen GWAS project | Male and female | 2018 |
| UNC5D | 3301 | 10,534,735 | European | IEU PDen GWAS project | Male and female | 2018 |
| ADAMTSs | 3301 | 10,534,735 | European | IEU PDen GWAS project | Male and female | 2018 |
| CD47 | 3301 | 10,534,735 | European | IEU PDen GWAS project | Male and female | 2018 |
| CUL4B | 3301 | 10,534,735 | European | IEU PDen GWAS project | Male and female | 2018 |
| DKK1 | 3301 | 10,534,735 | European | IEU PDen GWAS project | Male and female | 2018 |
| DnaJ | 3301 | 10,534,735 | European | IEU PDen GWAS project | Male and female | 2018 |
| CD39 | 3301 | 10,534,735 | European | IEU PDen GWAS project | Male and female | 2018 |
| ER | 3301 | 10,534,735 | European | IEU PDen GWAS project | Male and female | 2018 |
| PD | 482, 730 | 17,891,936 | European | IEU PDen GWAS project | Male and female | 2019 |
